# Implementation Methods and Research for a Post-truth World with Growing Inequities

**DOI:** 10.1007/s43477-022-00063-2

**Published:** 2022-12-27

**Authors:** John Øvretveit

**Affiliations:** Department of Learning Management Informatics and Ethics, Karolinska Institutet, and Research and Development Officer, Medical Management Centre, Stockholm Health Care Services, Stockholm, Sweden

**Keywords:** Anti-science, Post-truth, Story telling, Narrative methods, Implementation science, Quality improvement, Participatory research, Equity

## Abstract

The purpose of this article is to consider the changing context for implementation research and practice and new approaches which might now be more relevant for some implementation objectives. Factors that hindered implementation of evidence-based practices before the COVID-19 pandemic was an anti-science culture, strengthened by different media and appeals to emotion and identity. The article questions how effective are the rational-cognitive and individual models of change that frequency informs our research and practice. It describes challenges we face and considers methods we could use that might be more effective, including research-informed narrative methods, participatory research and practice, especially with culturally and linguistically diverse peoples, and adaptive implementation.

## Challenges

With our COVID-19 fatigue, and our wish to get back to “before times,” do we really appreciate what the accentuation of certain social trends over the last 3 years now means for implementation research and practice? The word of the year for Oxford Dictionaries in 2016 was “post-truth”, defined as, “relating to or denoting circumstances in which objective facts are less influential in shaping public opinion than appeals to emotion and personal belief” (Oxford Dictionaries, 2016). The credibility of scientific evidence may have declined for some sectors of the public during the COVID-19 pandemic, and an anti-science movement may have strengthened. Other recent events have changed our world and future: awareness of the impact of climate change, wars, economic recession and increasing poverty and inequities. This has changed the context for implementation and the attitudes and preoccupations of those who take up innovations. These include providers and the people they serve who are the intended ultimate beneficiaries of the innovation and of implementation. Also, service providers in all sectors are experiencing higher workload with increasing demand and shortages in staff, supplies and budgets. They are even less able to add implementation to their work that often involves changes to practice and adaptations which disrupt their every-day work, even though the innovation may save time in the future.

Implementers and implementation researchers are largely aware of these changes but have their methods and focus changed to be more relevant to this new world? This perspectives article describes some methods that could increase the effectiveness of implementation in the new world, including communication methods for behaviour change with designed storytelling, implementation partnerships and adaptive implementation. First, it considers some explanations and diagnoses for the reduced authority of science and decrease in respectful dialogue in some populations.

Key Points of the Article

- An anti-science movement, awareness of the impact of climate change, wars, economic recession and increasing poverty and inequities have changed the context for implementation.
- A story designed for behaviour change can be a powerful implementation strategy.
- Implementation partnerships are needed to design the right stories and implement research, but building partners’ trust needs more time and skills for implementers and researchers.
- Adaptive implementation can be carried out through quickly testing adaptions and can help make faster and more effective implementation in different situations.

## Post-truthism and Anti-science

One way to cope with fear and confusion is to rely more on the support of a group that shows a way forward, and to strengthen our sense of identity as a member of this group. Directional motivated reasoning, including cultural cognition, has been proposed whereby people compare their perceptions and opinions about science with their cultural, religious and/or political beliefs and values. Where there is a mismatch, evidence could be ignored or rejected to protect a person or groups worldview, especially in periods of heighted fear and confusion (Kahan et al., 2011). This could also apply to scientists who believe in evidence and in specific ways to make valid evidence. This could partially explain less willingness to listen to others about the validity of observational compared with experimental evidence and vice versa. Expression of more extreme views occurred in science during the COVID-19 pandemic, related also to the fast increase in rapid research and its publication, together with the lower validity of much research of both types, and less transparency about the limitations (Frampton et al., 2021; Ioannidis, 2022).

For some, trust in science and in healthcare was already damaged by unethical research and also personal experience of disrespectful treatment by providers (Scharff et al., 2010). Considerable research now shows that scepticism in some minority and marginalised groups about the scientific evidence they are presented with by scientists is sometimes well founded. The evidence may be poorly communicated, without the limitations made clear, or later confusingly changed (New York Times, 2021).

Disinformation and misinformation may become more widely believed, not only because of social media, but also because of growing of anti-science sentiment among some citizens, governments and policy makers. Knowledge of scientific methods and practice have long established that evidence is provisional and knowledge will change. It increasingly shows limitations to and uncertainties about this evidence, making it more difficult to identify valid and reliable evidence (Barzilai & Chinn, 2020; Kienhues et al., 2020). The COVID-19 pandemic revealed more flawed evidence, more scientific disagreement and more tendency for politicians to pick the evidence that supported their policy preferences than many of us recognised before the pandemic (Ioannidis, 2020).

One proposal of this article is that post-truthism and anti-science are just two subjects which implementers and researchers need to understand in order to develop methods more effective and relevant to the new world that we are living in. Cairney (2018) summarised policy research in a way which could challenge some implementation researchers’ assumptions about policy decision making, and also applies to service managers: “policymakers have many different ideas about what counts as good evidence; policymakers have to ignore almost all evidence and almost every decision taken in their name; policymakers do not control the policy process in the way that a rational policy cycle model suggests” (Cairney, 2018). For communicating research to policy makers, Carney proposes using knowledge from policy studies and psychology: “tell a story, since evidence will not speak for itself”, “form coalitions and provide evidence to empower your allies (don’t expect everyone to be influenced by the evidence)” and “be flexible, since a successful framing strategy in one venue may fail in another” (Cairney, 2018; see also Cairney & Kwiatkowski, 2017).

A related strategy is for researchers to interact more with policy makers to understand their perceptions and then shape their communication to their current concerns (Lomas, 2000). This strategy is also proposed for enabling uptake of evidence-based practices (EBPs) by healthcare professionals at the practice level (Rapport et al., 2021). Also, evidence often needs to be adapted and implemented in specific ways: co-design and participatory methods have been found to be effective, for example, to address vaccine hesitancy and equity issues (Wild et al., 2021).

The purpose of the article is not to suggest that implementation research should not use randomised controlled trials, observational or mixed methods and other traditional approaches: these have proven their value for certain objectives when well designed and conducted. Rather that the new world calls for us to make more use of three approaches that have already begun to be used in implementation research.

## Storytelling

Not just any story, but a story designed for behaviour change. Two effective communication strategies used by anti-science practitioners are anecdote and storytelling. For researchers, anecdote and storytelling are equated with invalid evidence and shunned in written reports and most scientific communications. But could implementation be helped by stories that influence people's hearts as well as their heads and that are designed to motivate and produce behaviour change, especially in our work with implementers and decision makers?

My personal experience is that stories are effective, if they are designed for the audience by using principles from research and practice in storytelling. My first awareness of the power of storytelling, not just for engaging audiences but also for behaviour change, was through my interest in religion and journalism. Stories have been used to communicate most faiths in the world for thousands of years and to motivate behaviour change, and continue to be used. Many journalist articles and media presentations start with the story of an individual’s experience and journey—journalism has been defined as “storytelling with a purpose” (Kovach & Rosenstiel, 2021).

Later I witnessed how effective storytelling was in my work evaluating HIV/AIDS programmes in Africa and the Zambia quality improvement reform (Bouchet et al., 2002; Chime et al., 2004). A small village on market day in Zambia in 1998. How does a public health prevention programme get villagers attention and motivate and teach them to follow HIV/AIDS prevention practices? Do we hand out written pamphlets which most cannot read? A small truck arrives and everyone abandons the market stalls to crowd around the truck. Out gets a group of young Zambians, who proceed to entertain the excited and cheering audience. They present carefully designed dramas about unfaithful husbands and the family coming down with “the sickness”, and songs about condoms and testing that the audience easily remembers, even though they have heard them many times before. They shout for more, but the truck has to leave to give similar entertainment to the staff of the local health centres, who are implementing innovations in care and service delivery models to provide newly needed services.

Why could I not use the same principles to engage and motivate the different managers and decision makers when we present the evaluation team’s report? I asked one on my African colleagues to tell the story of a married couple in one of the villages who contracted HIV/AIDS and died, leaving the grandmother to care for the three orphans as well as the four others she was already caring for. Then another team member presented a married couple who experienced the innovations we were evaluating. A third told the story of a nurse at a health centre who was enabled to implement the innovations and her views about how to implement it better. They explained that examples were based on what we had found in the evaluation interviews. Then we presented the quantitative data on the size of the challenge and the inequitable impact, and then the other findings with recommendations with costs and savings. At the meeting, and in my follow-up work, I found this had been a great success, and continued to use it in my other evaluations and reports in Africa. I started to use the same approach for presentations to non-academics in Sweden and elsewhere in my quality improvement and implementation studies. I only began to use this approach in journal reports for patient-focused journals, where it was accepted by peer reviewers which often included patients.

Part of my storytelling journey was following the growing research and reports on the subject, especially the recent neuroscience research and science-based guidance on systematic story development for different audiences (Martinez-Conde et al., 2019). I found that business leadership research had discovered that certain approaches to storytelling by leaders can be effective to help to implement the change strategies they are leading (“narrative leadership” Auvinen et al., 2013). A small industry has grown to teach leaders to “lead through stories” (Mckee & Fryer, 2003). The best simple guidance I found was the “and… but… therefore” structure (Olson, 2015) based on research reported in Olsen 2019. For science presentation, this structure is “[This is true and observable] and [this is true and observable OR this data exists], but [problem statement], therefore [this is how we are addressing the problem].” (Olson, 2019).

Some evidence suggests that stories, crafted in the right way and using the right story teller, connect with peoples’ emotions to communicate a need for change or an experience of change and can be part of a strategy for implementing change. Studies show that stories increase engagement when communicating science to non-expert audiences and increase people’s likelihood of remembering information (Dahlstrom, 2014; Graesser et al., 1980). One study found that, on average, facts were 20 times more likely to be remembered if they are part of a story (Murray, 2015).

My experience using this method in quality improvement is to ask a patient to a meeting of personnel, or use a video of a patient, and for the patient to recount their experience of their episode of care, describing delays, sub-optimal treatment and mistakes they noticed in and between different stages of their care. How would you feel if your loved one had this experience? Might any of our patients experience something like this? How might we know how many do have similar experiences? How much of this is caused by the system of care or because of carelessness by individual staff? These are four questions that I have found effective for motivating and committing staff to quality improvement implementation in the initial meetings, especially if combined with their peers describing their results using improvement and implementation methods.

Public health, inequity reduction, mental health and other programmes have used storytelling as part of their change strategies for some time (De Vecchi et al., 2016). Recently these methods have received discussion in implementation science. One conclusion of a review of storytelling interventions is that stories by patients, friends, family and caregivers are increasingly used to help uptake of research evidence (Brooks et al., 2022). The review also noted that the research rarely describes how stories are developed, or the theoretical assumptions or which approaches are effective for different objectives and target groups. The review usefully provides a research-informed framework to guide storytelling as an intervention for health-promoting behaviour change and which can be used by implementers for other changes.

My journey of discovery of effective implementation led to using two other approaches. These are also relevant to the new world, where faster and more equity-enhancing evidence-based implementation is increasingly required to meet the challenges experienced by service providers and ordinary people across the globe.

## Implementation Partnerships

Implementation partnerships are needed to design the right stories and implement research, but building partners’ trust needs more time and skills for implementers and researchers. In my evaluations of aid programmes with African colleagues, our field research had to begin with a visit to the tribal chief and village elders. I soon learned that this was not a quaint tradition, but an active element of a research partnership, and also prepared leaders to help the implementation of the recommendations.

Yes, there were ceremonies, but then, if the research team was judged to be people the chief and local elders wanted to work with, we would be invited to meet for some time with them to guide us in the research. Key to building trust was, first addressing a common fear that a programme would be stopped if we found it to be ineffective. I had learned not to accept contracts for evaluations where termination of the programme was an option, and to require a contract to specify that the purpose was to improve the programme and consider options for scale-up elsewhere. This is not possible for many evaluations. But explaining that the evaluation aimed to improve the aid programme was necessary to get both valid data and later implementation. If we were invited to meet with the chief and local elders, then we were able to learn about and discuss their local problems relating to the subject of the evaluation. We learned who were the key people to interview, and who could most help implement possible recommendations. This began trust building, and word was spread that the leaders had approved this team to talk to and work with.

As regards implementation, global health and public health have for some time used workers from the communities that they serve because, as they say, people like us, who speak like us are more trusted and influential especially for engaging difficult to reach communities. Recognising challenges in uptake of public health innovations by practitioners and communities, these fields have also used collaborative research approaches to understand and develop interventions and implementation strategies tailored for specific groups. One example is a recent project to communicate COVID-19 information to culturally and linguistically diverse (CALD) communities. This study shows how long-term trusting collaboration with these communities and their leaders with appropriate tailoring and delivery of communications using co-design ensures that health-related messages are not lost in translation (Wild et al., 2021).

One implication is that researchers, implementers and funders will need to give more resources and develop new skills for working more closely with both staff and end users of services, such as students, patients, clients and others. This is especially necessary if implementation is to contribute to reducing the inequities in access and use of effective services by historically marginalised groups (Goodyear-Smith et al., 2015). For example, in New Zealand, empowering indigenous Māori to take a governance role in the planning, development and execution of research as well as monitoring the project through its life cycle needs to be carried out by implementing innovations in ways which help groups to do this (Health Research Council, 2010).

## Adaptive Implementation

Adaptive implementation can be carried out through quickly testing adaptions and can help make faster and more effective implementation in different situations. My experiences in these African and similar international evaluation projects was of the advantages of providing feedback to implementers while the evaluation was being carried out. This third approach built on the partnership approach to both research and implementation, and grew out of my earlier work with quality improvement research and projects. I found that the early feedback approach outweighed the disadvantages of the feedback changing the innovation and implementation while it was being evaluated, if the study was a rapid evaluation of implementation. The general approach is now described and illustrated in five frameworks which are all variations of an iterative feedback approach (Øvretveit, 2022).

More recently the COVID-19 pandemic struck Stockholm. I was not prepared to continue my research which was unrelated to the crisis while my practising medical colleagues started working 14 h a day and my city was threatened. From previous research, we had built a partnership with the CEO of the primary and community care service delivery division in the region. Together we wrote an ethics application and flexible protocol for rapid implementation evaluation research, which was fast tracked, approved and started within 2 weeks. The programme so far has led to nine sub-projects and six peer reviewed publications, and reduced suffering and lowered costs through the faster and more effective implementation of management and organisation innovations (Ovretveit & Ohrling, 2021; Øvretveit, 2021).

There are a growing number of implementation researchers proposing that the speed of change calls for implementation science to increase its use and development of rapid impact research, especially for improving scale-up (Glasgow et al., 2014; Proctor et al., 2022). As well as longer-term traditional research designs such as cluster randomised, or stepped wedge designs, implementation science can make more use of adaptive implementation approaches. These implement innovations in different phases with adaptations in later phases. One example is a cluster randomised trial of an adaptive implementation strategy (Kilbourne et al., 2014). In this study, the initial implementation of an outreach innovation for veterans with serious illness found that some sites had made slow progress. The next phase of the study provided these sites with enhanced facilitation which then increased take up of the programme and helped understand some of the necessary conditions for implementing the innovation elsewhere.

Adaptive implementation is not only about researchers adjusting their design in relation to implementers’ progress but is also about adapting the innovation. Researchers study fidelity and adaption of innovations made by implementers, both intentionally or unintentionally and have developed frameworks to describe the adaptations and reasons for adaption (Rabin et al., 2018). These approaches help the effective adaptation of innovations and of implementation and cannot be achieved without the partnership approaches noted above. For implementers and researchers, new skills will be needed to use methods suited to the tasks and group. These cover the spectrum from consultation to co-design, which is more resource intensive and time consuming but often increases impact and may be less costly in the long run.

## Conclusion

This perspectives article proposed that implementation researchers and practitioners could make use of three approaches in some of their projects to make their work more relevant to the post-truth new world environment. Features of this new context that call for using these methods are the speed of change of the context for implementation, and the need to adapt implementation methods and innovations to the changing context. The article describes three approaches from the authors’ personal experience and through research that has used these approaches. These are designed storytelling for motivated behaviour change; beneficiary-led implementation partnerships and adaptive implementation. These approaches are particularly useful for developing implementation and innovations to decrease rather than increase inequities, which can happen inadvertently in some projects. Debate is welcome in GIRA and GISS about whether we can increase the impact and relevance of implementation in the new world by making more use of these and other approaches.

## Data Availability

No data were used for this article, apart from published research cited.
